# Functional Design and Biophysical Characterization of Analyte-Responsive Polymers

**DOI:** 10.1021/acs.biomac.5c00066

**Published:** 2025-07-25

**Authors:** Carolyn E. Curley, Katarina Jovic Dold, Jazmine A. Torres, A. Clay Richard, Eleenah Sanders, Jeffrey M. Halpern, Robert J. Pantazes, Eva Rose M. Balog

**Affiliations:** † School of Molecular and Physical Sciences, 6687 University of New England, Biddeford, Maine 04005, United States; ‡ Department of Chemical Engineering and Bioengineering, 3067 University of New Hampshire, Durham, New Hampshire 03824, United States; ∥ Department of Chemical Engineering, 1383 Auburn University, Auburn, Alabama 36849, United States

## Abstract

As a proof-of-concept for analyte-responsive polymers (ARPs) for biosensing, this study investigates how ligand binding changes the temperature-dependent dynamics and self-assembly of an elastin-like polymer (ELP) fused with a peptide recognition element for the small globular protein domain SH3. Using isothermal titration calorimetry, we characterized the apparent binding thermodynamics when one binding partner is fused with an ELP. Circular dichroism, dynamic light scattering, and temperature-dependent UV–vis spectroscopy were used to examine how ligand binding influences ARP conformational dynamics and phase behavior. SH3 binding was associated with an increase in transition temperature that reproduced in a complex medium and was consistent with predictions from a published model for ELP fusions. Addition of SH3 destabilized ARP assemblies, demonstrating that an ARP response can be specifically triggered by ligand binding. This work advances our understanding of how ligand binding and phase behavior are interdependent in systems involving intrinsically disordered proteins and their assemblies.

## Graphical abstract

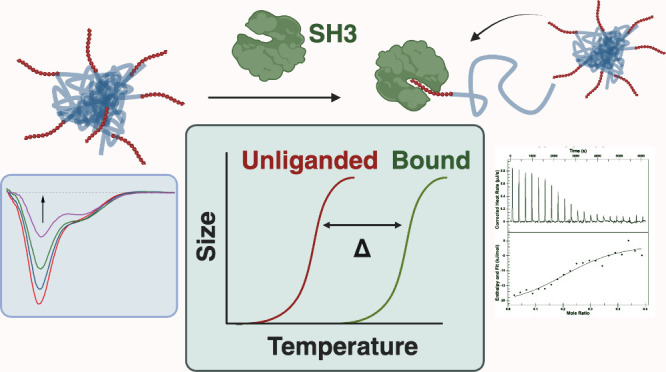

## Introduction

Engineered proteins ought to be ideal modular components for biosensors.^1–3^ Proteins evolved to specifically recognize and reversibly associate with their ligands. Ligand binding is associated with conformational changes in the protein.^4,5^ Ligand binding and conformational changes can occur at separate locations through allostery. These conformational changes can be transduced into other forms of energy and types of information.^6–8^

Generalizable allosteric protein biosensors exist.^9–13^ Some depend on the dramatic conformational switching behavior of the calmodulin domain.^9,14,15^ Others have used a multicomponent cage, latch, and key strategy where analyte binding is coupled to luminescence.^10,16^ Multicomponent optical systems are one solution to the challenge of identifying analyte-binding domains that undergo significant conformational changes coupled to a sensitive output. These systems have limitations; for one, having multiple components adds complexity. A label-free approach would also be advantageous to simplify preparation and preserve native biomolecular structure and function.^17^

Elastin-like polymer (ELP) fusions present a possible solution to these challenges. Allosteric regulation of the phase transition of ELPs has been demonstrated using calmodulin and a calmodulin-derived peptide.^18–20^ The transition temperature (*T*_*t*_) of an ELP fusion can be tuned by the character of the solvent-exposed surface area of the fusion partner.^21,22^ Ligand-responsive ELPs that self-assemble into defined architectures in the presence of a protein or small molecule have been developed for affinity precipitation and synthetic sensory protocells.^23,24^ Previous studies have also demonstrated that ligand binding can promote disassembly of polymer-based nanocontainers, driven by shifts in the hydrophilic–lipophilic balance upon ligand binding.^25^ In a different approach, hybrid bioconjugates of ELPs and oligonucleotides have been engineered to form thermoresponsive micelles capable of specific protein interactions without disrupting micellar structure.^26^ ELPs can be end-tethered to an electrochemical sensor surface, where their reversible conformational changes in response to stimuli can be detected as changes in impedance.^27^ Together, these facts make ELP fusions a compelling framework for investigating polymer systems that respond dynamically to ligand binding and promising components for analyte-responsive elements in label-free electrochemical biosensors.

As proof-of-concept for the application of ELP fusions as analyte-responsive polymers (ARPs), we designed and characterized a model system to test whether ligand binding could produce a shift in transition temperature (Δ*T*_*t*_) without relying on a dramatic conformational change such as that of calmodulin (Figure 1A). The Src homology 3 (SH3) domain is a small adaptor domain found in signaling proteins throughout eukaryotic proteomes.^28^ SH3 is an ideal model analyte because its binding has been extensively characterized structurally and thermodynamically.^29–33^ This allowed us to investigate how a binding interaction changes when one binding partner is placed in the context of an ELP fusion. This investigation is worthwhile for its contributions both to the development of engineering principles for ARP biosensors and related stimuli-responsive biomaterials and to our understanding of protein–protein interactions in the context of intrinsically disordered proteins and biomolecular condensates.

**Figure 1:**
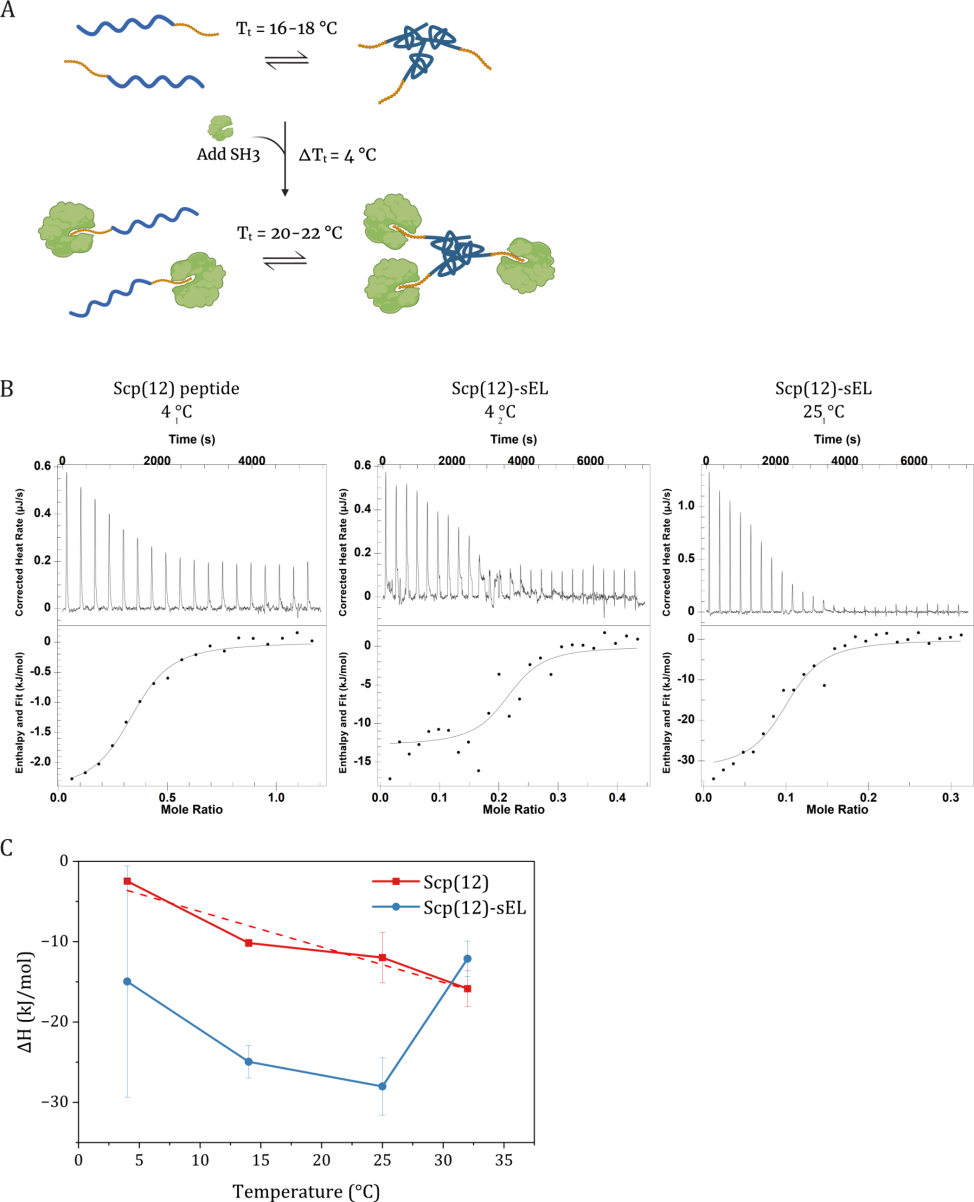
The peptide-ELP fusion Scp(12)-sEL binds to Abp1p SH3. (A) Model of the ARP system. Created in BioRender; Curley, C. E. (2024) https://BioRender.com/w72q216. (B) Representative binding isotherms from ITC experiments. (C) Temperature dependence of the apparent binding enthalpy for Scp(12) and Scp(12)-sEL binding to SH3 allows the calculation of constant pressure heat capacity.

## Experimental (Materials and Methods)

### Molecular Cloning

Circular polymerase extension cloning^34^ was used to introduce the sequences encoding the Scp(12), Sjl, and Prk peptides into the template plasmid POE sEL (expression construct originally described in Ghosh et al. as “ELP-1”).^35^ DNA encoding each peptide sequence was included within the corresponding forward primer for amplification of the insert. All PCR steps used Q5 polymerase (New England Biolabs, Ipswich, MA, USA) and those requiring amplification of sEL regions used GC enhancer due to its highly repetitive, GC-rich structure. Successful assembly of all plasmids was confirmed by Sanger sequencing (Eurofins Genomics, Louisville, KY, USA). Full DNA, peptide, and protein sequence information is provided in Tables S1–S3.

To make pET28a SH3, a MiniGene encoding *Saccharomyces cerevisiae* Abp1p SH3 Pro535-Asn592 flanked with NheI and BamHI restriction sites was synthesized and delivered in an IDT-Kan vector (Integrated DNA Technologies, Coralville, IA, USA). Recipient vector was prepared by digesting pET28a Cas9-Cys (Addgene plasmid #53261) with NotI to excise the Cas9 insert, isolating the vector from the insert using gel purification, and religating the empty vector. The SH3 insert was then subcloned into “empty” pET28a using NheI and BamHI cloning sites. Substitution of the conserved tryptophan 569 in the peptide-binding pocket with an alanine abolishes most binding to standard ligands of Abp1p SH3.^36^ The plasmid pET28a SH3^W58A^ (numbering specific to our SH3 expression constructs) was purchased from Twist Bioscience (South San Francisco, CA, USA).

### Protein Expression and Purification

To produce ELPs (Scp(12)-sEL, Sjl-sEL, Prk-sEL, and K-sEL), chemically competent BL21­(DE3) *Escherichia coli* (New England Biolabs, Ipswich, MA, USA) were transformed with the appropriate expression plasmid and plated on 2XYT agar +100 μg/mL carbenicillin medium. Transformants were grown overnight at 37 °C and then inoculated into starter cultures of SuperBroth +100 μg/mL carbenicillin. Starter cultures (25 mL) were grown at 37 °C while shaking at 200 rpm for 4 h. The growth was then scaled by adding the entire volume of starter culture to 1 L of SuperBroth +100 μg/mL carbenicillin. The 1 L cultures continued growing at 37 °C while shaking at 200 rpm for 24 h without induction. Cells were harvested by centrifugation (4200*g*, 4 °C, 20 min). Periplasmic extraction and purification by iterative temperature cycling were performed as previously described in detail in an open-access protocol.^37^

To produce wildtype and W58A mutant Abp1p SH3 domains, chemically competent BL21­(DE3) *Escherichia coli* (New England Biolabs, Ipswich, MA, USA) were transformed with the appropriate expression plasmid, and cells were plated on 2XYT agar +50 μg/mL kanamycin medium. Transformants were allowed to grow overnight at 37 °C and then were inoculated into 25 mL starter cultures of 2XYT + 50 μg/mL kanamycin. Starter cultures were grown at 37 °C while shaking at 200 rpm for 3 h. The entire starter culture was then added to 1 L of 2XYT + 50 μg/mL kanamycin to scale the expression. The 1 L cultures continued growing at 37 °C and shaking at 200 rpm until the OD at 600 nm reached 0.6. The cultures were then induced with 1 mM IPTG and grown overnight at 22 °C while shaking at 200 rpm. Cells were harvested by centrifugation (4200g, 22 °C, 20 min). Cells were lysed with B-PER Complete Bacterial Protein Extraction Reagent (Thermo Fischer Scientific, Waltham, MA, USA) in the presence of protease inhibitors and purified by immobilized nickel affinity chromatography utilizing the histidine-tag genetically engineered to the N-terminus of the SH3 domain. Protein was eluted with 25 mM Tris-HCl, 250 mM NaCl, 400 mM imidazole, pH 8.0, and immediately dialyzed into 50 mM sodium phosphate, 100 mM NaCl, 10% glycerol, pH 7.0, while stirring overnight at 4 °C. Aliquots were flash-frozen in liquid nitrogen and stored at – 80 °C.

Purified proteins were characterized by SDS–PAGE (Figure S1) using Bio-Rad Precision Plus Protein Dual Xtra-Prestained Protein Standards. All proteins except K-sEL were visualized using Coomassie-based stain; K-sEL was visualized using silver stain (FASTSilver, G-Biosciences). The identity and integrity of Scp(12)-sEL were confirmed by electrospray ionization mass spectrometry (ESI-MS) on a Waters Synapt G2-Si HDMS.

### Isothermal Titration Calorimetry

Binding experiments were carried out at 4, 14, 25, and 32 °C using an Affinity ITC instrument (TA Instruments, New Castle, DE, USA). All experiments were performed in 50 mM sodium phosphate, 100 mM NaCl, and 10% (v/v) glycerol, pH = 7.0, with a stir rate of 125 rpm and replicated at least two times. Injection sizes ranged from 1.25–2.5 μL with an injection rate of 0.5 μL/s and 300 s intervals between injections. The baseline was allowed to auto equilibrate, and a 60 s initial baseline was recorded before the first injection.

Peptide and SH3 experiments had concentration ranges of 0.90–1.50 mM for the peptide and 0.075–0.200 mM for SH3. SH3 and ELP binding experiments had concentration ranges of 0.230–0.450 mM for SH3 and 0.100–0.175 mM for the ELP. Baselines were generated automatically by NanoAnalyze and corrected manually. Injection peaks without a stable baseline were discarded from the isotherm. To account for heat of dilution, the average area of 5–8 blank injections of syringe content into buffer under identical conditions as each experiment were subtracted from each isotherm. The isotherms were then fitted using an independent model in NanoAnalyze.

### Circular Dichroism

SH3 binding experiments were performed with Scp(12)-sEL and K-sEL that were kept at a constant concentration (10 μM), while the SH3 concentration was varied (30–60 μM). Each protein was also measured alone, including the SH3 samples, at different concentrations. SH3 binding to Scp(12)-sEL (K-sEL) was assessed at 4 different ratios: 3:1, 4:1, 5:1, and 6:1. All CD samples were prepared in 50 mM sodium phosphate, 100 mM NaF, pH 7.0, and kept on ice. Lyophilized Scp(12)-sEL and K-sEL were reconstituted in this buffer, and SH3 was buffer exchanged into it using Zeba 7K desalting columns (Thermo Fisher Scientific), following the manufacturer’s recommendations. The CD measurements were performed using a JASCO J-1500 CD spectrometer equipped with the Peltier cell, in a 1 mm path length cuvette (Starna). Temperature Interval Measurement software (Spectra Manager, JASCO) was used to collect the spectra at 10 °C intervals, from 4 to 34 °C, and then back to 4 °C. Only the heating temperature ramp data are shown in the present work; however, the cooling temperature ramp confirmed the reversibility of the ELP temperature response in all instances. Samples were equilibrated for 2 min at each temperature before the first of five spectra was recorded at a given temperature. Both the heating and cooling ramps were set to 5 °C/min. The general CD spectrometer parameters were as follows: wavelength range 180–275 nm, data pitch 0.2 nm, and D.I.T. (digital integration time) 4 s, bandwidth 8 nm, and scanning speed 100 nm/min. The instrument was purged with ultrapure liquid nitrogen before and during operation at a flow rate of 50 SCFH (standard cubic feet per hour).

### UV–Visible Spectroscopy

Protein solutions were prepared on ice in 50 mM sodium phosphate and 200 mM NaCl, pH 7.0. Absorbance at 400 nm (OD_400_) was measured using an Olis computerized Hewlett-Packard 8452a diode array spectrophotometer equipped with a qX2 air cooled, Peltier-controlled cuvette holder from Quantum Northwest. Temperature ramping experiments were performed at a ramp rate of 1 °C/min in a low volume quartz cuvette, and absorbance spectra from 390 to 500 nm were collected every ∼15 s. For experiments in cell culture medium, ELPs were dissolved on ice in freshly prepared, 0.2 μm sterile filtered MEMα (GIBCO 12-571-071) supplemented with 10% fetal bovine serum (premium, Gibco A5670701).

### Dynamic Light Scattering

DLS measurements were performed on a Malvern Zetasizer Ultra Red Label. ELP samples were prepared by dissolving lyophilized protein in a prechilled, filtered (0.22 μm) solvent on ice for at least 10 min and gently vortexed to ensure complete dissolution. Typical concentrations were 0.3–0.35 mg/mL (∼20 μM); all Δ*T_t_* comparisons were made controlling for sample ELP concentration. Samples containing SH3 were prepared by the dilution of a purified concentrated stock of SH3 on ice. For SH3-ELP complex samples, lyophilized ELP was dissolved in a solution containing 92–163 μM (∼4× to 7× molar excess) SH3 for at least 10 min on ice, vortexed, and filtered through a 0.22 μM syringe filter before analysis. Measurements were made in a low-volume quartz cuvette (ZEN2112) at a scattering angle of 173 ° using a He–Ne laser with λ = 633 nm. Solvent blank and polystyrene bead standard measurements were performed prior to each experiment. For temperature-ramp experiments, samples were allowed to equilibrate at each temperature for 120 s before triplicate size measurements at each 2 °C increment. Each temperature ramp was performed at least twice per sample. The majority peak was chosen from the volume particle size distribution, and the hydrodynamic diameter was assigned using the intensity particle size distribution. Plot data points represent average values ± the standard deviation. For multiangle dynamic light scattering (MADLS) measurements, triplicate measurements at 12°, 90°, and 173° were performed at each temperature in disposable 10 × 10 plastic cells (DTS0012). For SH3 titration experiments, Scp(12)-sEL was dissolved to a concentration of ∼0.3 mg/mL in cold, sterile-filtered 50 mM sodium phosphate and 100 mM NaCl, pH = 7.0. Size measurements were performed in triplicate at each temperature in 2 °C increments with no extra equilibration time. Following observation of a transition beginning at 10 °C (Figure S2), samples were held at 16 °C for titration with either SH3 or buffer. SH3 (163 μM) was added in increments of 3.08 μL to deliver 0.25x mol equiv of SH3 at a time with a minimum of volume change. For the buffer experiment, paired volumes were added. Initial analysis was performed using ZS EXPLORER software version 3.2.1.11, and further analysis and plotting were performed in Origin 2024.

### Computational Structure Prediction and Property Calculation

No experimental structures of the Scp(12), Sjl, or Prk peptides alone or in complex with SH3 have been published. Therefore, they were computationally predicted in this study to allow for prediction of Δ*T_t_* using the model of Christensen et al. Protein Data Bank (PDB)^38^ file 2RPN^39^ was selected as a template structure. This file contains the NMR-determined structure of SH3 in complex with an 18 amino acid peptide (ArkA) from actin-regulating kinase 1 (Ark1p) from *Saccharomyces cerevisiae*. Model no. 1.1 was arbitrarily selected as the template for this work from the 20 conformations included in the PDB file.

The experiments in the present work used an 81 amino acid (AA) version of SH3 engineered to provide an N-terminal affinity tag and a thrombin protease cleavage site. Residues 2–59 (of 59) from 2RPN matched the 58 C-terminal residues of the experimental sequence used herein. RosettaFold^40^ was used to predict the structure of the 81 AA version of SH3. This structure was then superimposed onto the template structure of SH3 from 2RPN using UCSF ChimeraX,^41^ creating a computationally predicted structure of SH3 in complex with the experimental Ark1p peptide. Sequence alignments of Ark1p with Scp(12), Sjl, and Prk were manually generated (Table S4). Based on these sequence alignments, point mutations were introduced using the “swapaa” command of UCSF ChimeraX and extra residues at the ends of the peptides were deleted. Each complex underwent a two-stage energy minimization: a CHARMM36 ^42^ energy minimization with a harmonic constraint on backbone atoms of 10 kcal/(mol Å^2^) followed by a Rosetta^43^ all-atom energy minimization. The use of CHARMM followed by Rosetta is consistent with prior computational studies we have conducted,^44–47^ and was done because it is our experience that CHARMM is best able to resolve structural clashes while Rosetta is the most widely used protein design force field.

The solvent accessible surface area was calculated using the rolling sphere approach proposed by Lee and Richards^48^ and refined by Shrake and Rupley.^49^ Briefly, the spherical surface of each atom is discretized into a Fibonacci lattice where, starting from the bottom, each successive point increases in azimuth according to the golden ratio and the polar angles are distributed evenly, ensuring efficiency and a uniform distribution of points on the sphere. The radius of the surface is the van der Waals radius plus a water probe (1.4 Å). As per Shrake and Rupley, the spheres were represented by 92 test points (i.e., the “dots” required to calculate SI_C_ for the Christensen et al. Δ*T*_*t*_ model). The default 92 dots from Shrake and Rupley were used here because we were unable to determine the number used by Christensen et al., although we believe they used more than 92 based on an analysis of their images. Each point on the surface of the sphere is checked with its nearest neighboring atoms to determine whether it is exposed to solvent or buried. The number of points that are exposed are then used to calculate the solvent exposed surface area of that atom, residue, protein or peptide. Charged solvent accessible surface areas were calculated using (1) only the peptide atoms as buriers and (2) all SH3-peptide complex atoms as possible buriers. Figure 5 illustrates the predicted Scp(12)-SH3 complex as both a space-filling model and using the dots required to calculate SI_C_.

The difference in the fraction of charged dots versus the fraction of the charged solvent accessible surface area reported in Table 2 is due to the details of this calculation. Every atom has 92 dots assigned to it and only exposed dots are used to calculate surface area. However, the amount of surface area attributed to each dot is dependent on the diameter of the atom: atoms with smaller diameters have less surface area per dot than atoms with larger diameters.

## Results and Discussion

The SH3 domain from *Saccharomyces cerevisiae* Abp1p binds proline-rich (PXXP-like) motifs with a range of affinities.^39^ We quantified the binding of recombinant SH3 to the peptide Scp(12) and a candidate ARP, ELP fusion Scp(12)-sEL, using isothermal titration calorimetry (ITC) (Figure 1B, Table 1, Figure S3). By performing ITC at different temperatures, we determined binding heat capacities. Changes in binding heat capacity can indicate additional equilibria coupled with ligand binding.^5^ The *K*_d_ of SH3 for the Scp(12) peptide ranged from 4.2 to 35 μM, typical of moderate-affinity interactions observed for SH3 domains. While there was no clear trend in *K*_d_ with temperature, ΔH became more negative with increasing temperature, suggesting coupling between ligand binding and conformational change (i.e., induced fit). The slope of SH3-Scp(12) peptide binding enthalpy gives an apparent binding heat capacity of Δ*C*_*p*_ = −0.44 kJ/(mol K).

**Table 1:** Thermodynamic Binding Parameters of SH3 to Peptide and Peptide-ELP Ligands^a^

sample	*T* (°C)	*K*_d_ (×10^–6^ M)	–ΔG (kJ/mol)	–ΔH (kJ/mol)	–*T*Δ*S* (kJ/mol)	*n* ^b^
Scp(12)	4	4.2 ± 0.1	28.5	2.5	–26.0	0.41
	14	35.3 ± 4.4	24.5	10.2	–14.3	0.50
	25	19.4 ± 13.0	27.6	12.0	–15.6	0.47
	32	28.8 ± 10.6	26.8	15.8	–11.0	0.37
Scp(12)-sEL	4	1.08 ± 0.54	32.5	15.0	–16.9	0.14
	14	7.33 ± 4.78	28.5	24.9	–3.6	0.29
	25	0.422 ± 0.070	36.4	28.0	–8.4	0.12
	32	3.13 ± 3.14	32.6	12.1	–20.9	0.18
K-sEL	25	NB				
Prk	32	0.81 ± 0.03	35.6	18.1	–17.5	0.38
SH3^W58A^ + Prk	32	NB				

a NB = no binding. K-sEL is a negative control ELP lacking an SH3-binding motif. Prk is a positive control for strong SH3-peptide binding. SH3W58A is a nonbinding SH3 negative control.

b *n* values provided represent peptide:SH3 for Scp(12) and Prk experiments and SH3:ELP for K-sEL and Scp(12)-sEL experiments.

SH3 bound Scp(12)-sEL with an apparent *K*_d_ ranging from 0.42 to 7.3 μM. When interpreting these values, it is important to consider the additional complexity of this system. The equilibrium of ELP self-assembly is highly temperature dependent; even when *T* < *T*_*t*_, ELPs tend to oligomerize. The increased apparent affinity may be due to avidity effects, a hypothesis supported by the consistently lower ITC *n* value with Scp(12)-sEL compared with Scp(12), which is determined from the Mole Ratio. In cases where the measured concentrations of the titrant and titrand are accurate and both components are 100% active, the ITC *n* value is equivalent to the binding stoichiometry. A lower *n* value correlates with fewer ligands (titrant) bound per macromolecule (titrand) in solution. It may also reflect a smaller active fraction of titrand, producing a lower effective concentration of accessible macromolecules. To address potential causes, we performed high-definition mass spectrometry to characterize the intact molecular weight of Scp(12)-sEL (Figure S4), ruling out truncation or degradation of the SH3 binding motif. Another possibility is that when Scp(12)-sEL undergoes coacervation, some SH3 binding sites become inaccessible. Taken together, the higher affinity and lower *n* suggest that a fraction of Scp(12)-sEL may be in a coacervate phase while associating with SH3. In this state, solvent-accessible Scp(12) motifs contribute to avidity effects, while coacervation limits the available binding sites.

The apparent heat capacity of Scp(12)-sEL binding to SH3 showed a distinct temperature dependence compared to that of Scp(12) (Figure 1C). Between 4 and 25 °C, Δ*H* and temperature exhibited the same constant negative relationship, with a similar heat capacity (Δ*C_p_* = – 0.42 kJ/(mol K)). This trend reversed at 32 °C, indicating the contribution of a separate, temperature-dependent equilibrium to the apparent enthalpy of binding. We hypothesize that this reflects endothermic loss of hydrophobic hydration associated with ELP coacervation.^50,51^

We investigated how SH3 binding influenced the secondary structure and temperature response of Scp(12)-sEL by using circular dichroism (CD). Like other ELPs, Scp(12)-sEL and K-sEL display a characteristic, reversible change in CD signal with temperature (Figure 2A). Specifically, a negative band at 198 nm is a signature of disorder and decreases in amplitude with increasing temperature, while positive ellipticity at 206–212 nm is associated with type II β-turns.^52,53^ The CD spectrum of SH3 remains constant across the temperature range studied (Figure S5). The Scp(12)-sEL spectrum maintained the same overall peak profile at all temperatures, while the K-sEL peak profile noticeably changed at 34 °C. We hypothesize that this reflects a higher propensity of K-sEL toward intramolecular self-association, leading to ordering, while the presence of positively charged groups on the termini of Scp(12)-sEL leads to extension and intramolecular repulsion, a hypothesis supported by our DLS data.

**Figure 2:**
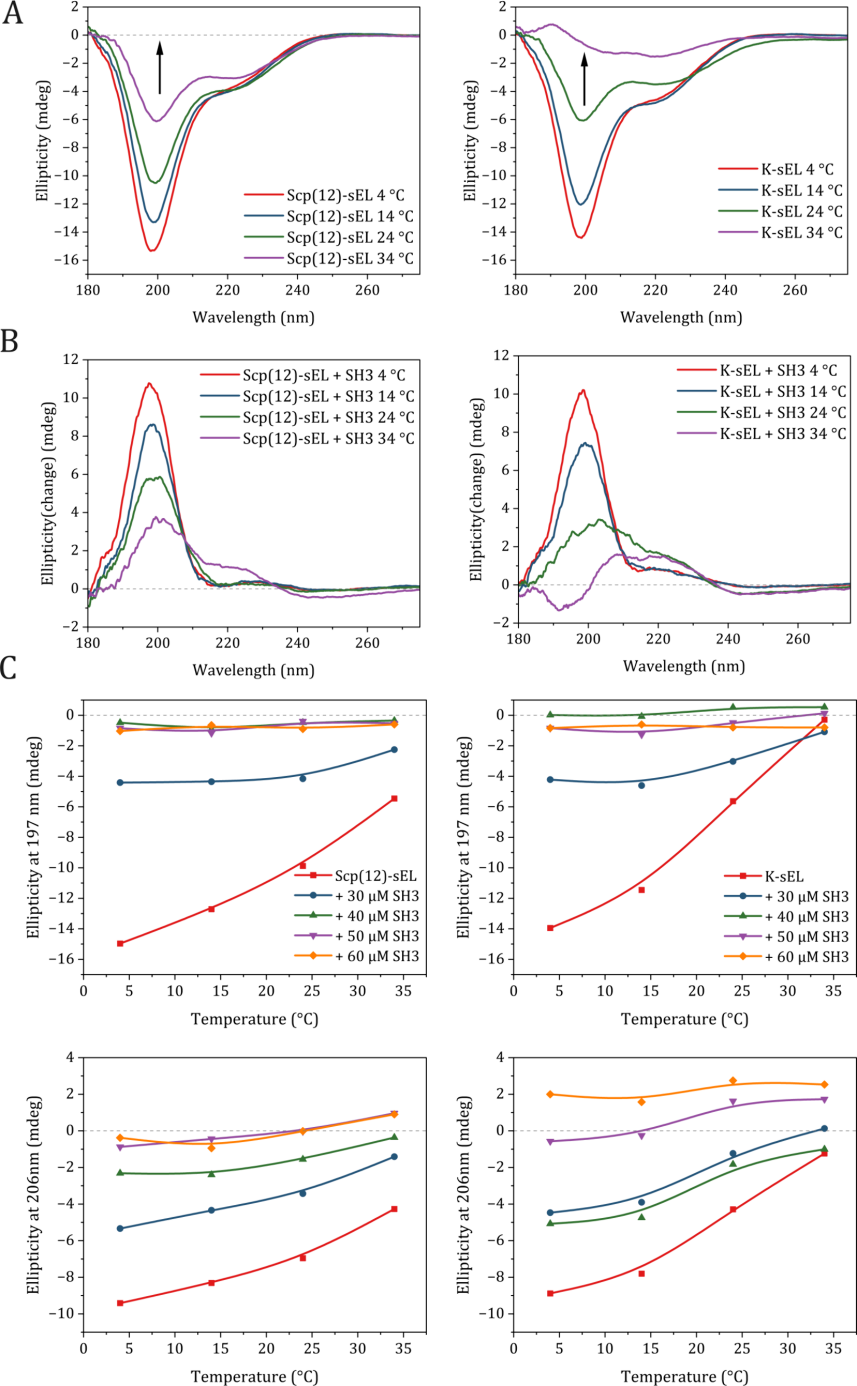
Structural responses of Scp(12)-sEL and K-sEL to SH3 binding. (A) CD spectra of Scp(12)-sEL and K-sEL (10 μM). (B) Change in the ellipticity with SH3 (30 μM). (C) Ellipticity as a function of temperature for characteristic peaks associated with the conformational transition from random coil to β-turn structures.

To isolate spectral features representative of structural changes in Scp(12)-sEL or K-sEL in the presence of SH3, spectra of individual protein components were subtracted from the spectrum of the mixture (Figure 2B):

$${\mathrm{Ellipticity}}_{\mathrm{change}}={\mathrm{Ellipticity}}_{\mathrm{Scp}\left(12\right)\mathrm{‐sEL}+\mathrm{SH}3\left(\mathrm{K‐}\mathrm{sEL}+\mathrm{SH}3\right)}-{\mathrm{Ellipticity}}_{\mathrm{SH}3}-{\mathrm{Ellipticity}}_{\mathrm{Scp}\left(12\right)\mathrm{‐sEL}\left(\mathrm{K‐}\mathrm{sEL}\right)}$$

The most significant binding-induced structural changes are increased ellipticity at 198 nm, representing increased order. These changes are most pronounced at 4 °C. One interpretation is that SH3 binding increases the order for free, monomeric Scp(12)-sEL. At higher temperatures, where more Scp(12)-sEL has collapsed and/or aggregated, no further ordering occurs upon binding. Another interpretation is that a less dramatic structural change with SH3 binding is observed at elevated temperature because the fraction of Scp(12)-sEL available for binding is reduced.

We selected two wavelengths diagnostic of ELP disorder and order (198 and 206 nm) to monitor changes in the secondary structure with temperature and SH3 concentration (Figure 2C). With SH3, Scp(12)-sEL shows reduced temperature sensitivity, suggesting that SH3 binding has a stabilizing effect. The ellipticity changes with increasing SH3 concentration appear to plateau, consistent with saturation binding. In contrast, K-sEL maintains temperature sensitivity in the presence of SH3, and its ellipticity changes do not plateau with increasing SH3 concentration, indicating nonspecific interactions rather than saturation binding.

We used dynamic light scattering (DLS) to investigate whether SH3 binding shifts the *T*_*t*_ of Scp(12)-sEL. Individual proteins were evaluated using multiangle dynamic light scattering (MADLS) to ensure accurate peak assignments in complex polymodal samples (Figure S6). SH3 exhibited a major peak with a hydrodynamic diameter (*D*_h_) of 4–6 nm. Scp(12)-sEL displayed multiple populations, likely representing monomers (15–30 nm) and nucleation phase precursors to phase separation (∼70–200 nm) below *Tt*, and larger aggregates (500–1200+) above the *T*_*t*_. K-sEL appeared as monomers (9–15 nm) below *T*_*t*_ and larger aggregates (500–2000 nm) above *T*_*t*_, but lacked detectable intermediate oligomers. To quantitatively compare conditions, we tabulated the temperature at which particle sizes exceeded a defined threshold, usually 200 nm (Table S5). However, given the complexity of the multimodal particle distributions, the different assembly behaviors from one ELP to the next, and the potential influence of SH3 on solubility, oligomerization, and assembly dynamics, these *T*_*t*_ values are a practical but simplified metric intended to capture trends across conditions that may differ in their underlying behavior.

In the absence of SH3, Scp(12)-sEL has *T*_*t*_ = 16–18 °C (Figure 3A). In the presence of SH3, the *T*_*t*_ shifts +4 °C to 20–22 °C. When SH3^W58A^ was used, the *T*_*t*_ of Scp(12)-sEL did not increase (16 °C). Likewise, the *T*_*t*_ of K-sEL (26 °C) did not increase with SH3 addition. Taken together, these results support the hypothesis that SH3 binding causes the observed change in the *T*_*t*_ of Scp(12)-sEL in the presence of SH3.

**Figure 3:**
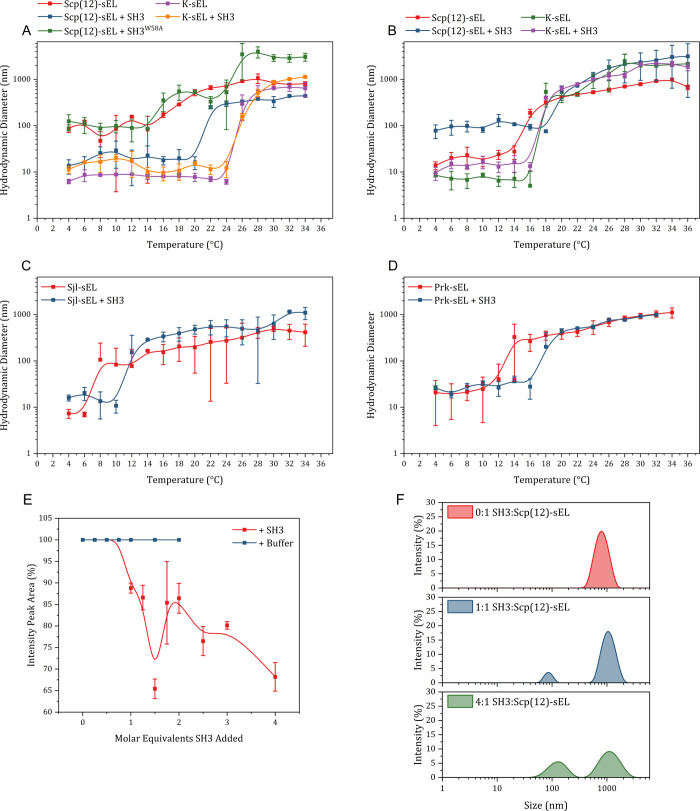
DLS reveals shifts in transition temperature with SH3 binding. (A) Temperature-dependent DLS of Scp(12)-sEL and K-sEL (20 μM) with and without SH3 or SH3^W58A^ (92–163 μM) in 50 mM sodium phosphate and 100 mM NaCl, pH 7.0. (B) Same as (A) but with 200 mM NaCl. (C) Temperature-dependent DLS of Sjl-sEL with and without SH3. (D) Same as (C) with Prk-sEL. (E) Titration of SH3 into Scp(12)-sEL coacervate (20 μM) while monitoring area percent of the intensity peak representing micrometer-scale phase separation. (F) Intensity peak distribution of Scp(12)-sEL during SH3 titration.

Under more stringent binding conditions (200 mM NaCl), we again measured a Δ*T*_*t*_ = +4 °C for Scp(12)-sEL with SH3 and no change for K-sEL (Figure 3B). The −2 °C shift in the Scp(12)-sEL curves and –8 °C shift in the K-sEL curves imply that ligand- and salt-responsiveness coexist within this polymer system and that differential salt-responsiveness based on hydrophobic surface area is preserved.

The Δ*T*_*t*_ effect was replicated with two different ARPs, Sjl-sEL and Prk-sEL (Figure 3C,D). That Δ*T*_*t*_ = +4 °C in each case suggests that Δ*T*_*t*_ may be determined more by the nature of the ligand than affinity. Notably, the addition of SH3 to Scp(12)-sEL after coacervation induced an isothermal shift in *T*_*t*_ in a concentration-dependent manner (Figure 3E,F).

We next used UV–vis turbidity measurements at 400 nm (OD_400_) to confirm the observed effects on Δ*T*_*t*_ with a standard and independent optical technique. Scp(12)-sEL showed a Δ*T*_*t*_ ≈ +8 °C with added SH3, while the control ELP K-sEL did not (Figure 4A). With increasing concentrations of Scp(12)-sEL at constant SH3, the +Δ*T*_*t*_ effect was diminished (Figure 4B), suggesting that saturating ligand binding is important for producing a clear shift in phase behavior, consistent with the SH3 titration DLS results above. Notably, Scp(12)-sEL exhibited a nonlinear relationship between concentration and *T*_*t*_, possibly due to the polymer’s net positive charge promoting interchain repulsion that opposes assembly. Supporting this hypothesis, the presence of SH3 restored the expected trend of decreasing *T*_*t*_ with increasing ELP concentration, potentially by balancing positive and negative charges upon binding despite increasing the total charged surface area.

**Figure 4:**
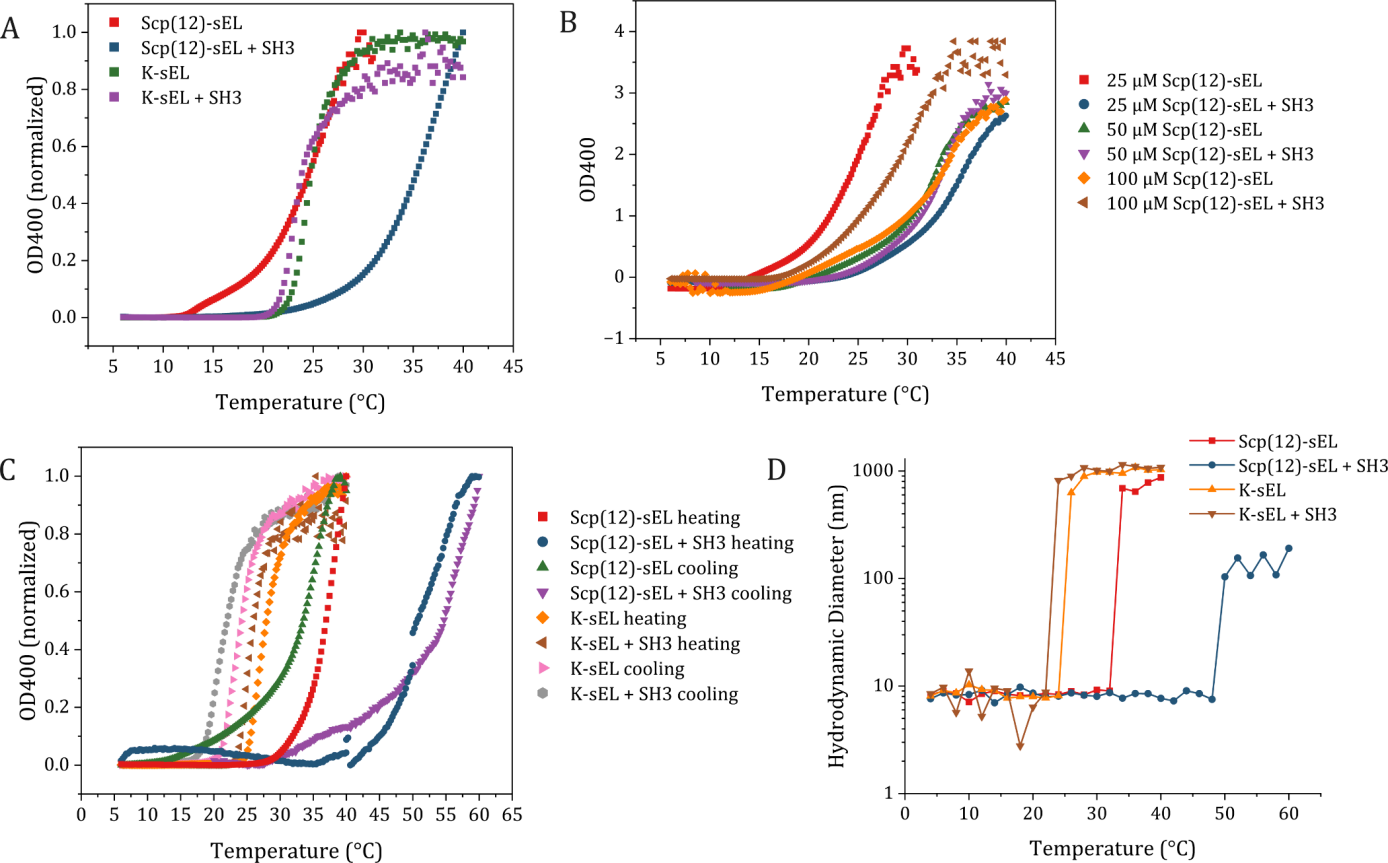
Temperature-dependent turbidity profiles of ELPs show ligand-responsive behavior. (A) Temperature-dependent UV–vis absorbance (OD400) of Scp(12)-sEL and K-sEL (25 μM) with and without SH3 (163 μM) in 50 mM sodium phosphate and 200 mM NaCl, pH 7.0. (B) OD400 measurements of increasing concentrations of Scp(12)-sEL with and without SH3 (163 μM) in the same buffer conditions. (C) Heating and cooling experiments with Scp(12)-sEL and K-sEL (25 μM) with and without SH3 (163 μM) in MEMα + 10% FBS mammalian cell culture medium. Note that the heating curve for Scp(12)-sEL + SH3 was acquired in three sequential runs (6–40 °C, 40–50 °C, and 50–60 °C) due to an unexpectedly high transition temperature, resulting in a discontinuous appearance of the data. (D) DLS measurements of samples in MEMα + 10% FBS corroborate UV–vis *T*_*t*_ results.

To test whether Scp(12)-sEL remained ligand-responsive in more physiologically relevant conditions, we repeated the experiment in mammalian cell culture medium (MEMα) supplemented with 10% fetal bovine serum (FBS), a complex mixture containing over 1,000 components including growth factors and other plasma proteins, lipids, carbohydrates, and minerals. In these conditions, Scp(12)-sEL showed a more dramatic Δ*T*_*t*_ = +16 °C with SH3 (Figure 4C), while again the control ELP K-sEL did not change with SH3. These findings were confirmed with DLS measurements of the same samples (Figure 4D). Given the complexity of the solution, it was not possible to distinguish a nanometer-scale peak corresponding to soluble ELP monomers. Figure 4D plots the size of the majority peak by volume, which at lower temperatures is likely an abundant component of the medium such as BSA. At higher temperatures, however, all solutions displayed distinct temperature-dependent formation of larger particles, consistent with ELP assembly. Cooling experiments were performed to test the reversibility of this assembly behavior and to control for the possibility that we were observing precipitation of medium components. For both Scp(12)-sEL and K-sEL, assembly was reversible with some hysteresis and maintained the same Δ*T*_*t*_ response in the presence of SH3.

Christensen et al. proposed a linear model (*R* ^2^ = 0.93) for predicting the Δ*T*_*t*_ of ELP fusion proteins based on the charged surface index (SI_C_), which is related but not identical to the percentage of solvent accessible surface area (SASA) of charged residues.^22^ Structures of Scp(12), Sjl, and Prk alone and in complex with SH3 were computationally predicted and used to calculate SI_C_ values, which were then used to predict Δ*T*_*t*_ values (Figure 5, Table 2). For Scp(12) peptide fusion, Christensen et al.’s model predicted a Δ*T*_*t*_ compared to K-sEL of nearly −20 °C, approximately twice the experimental result (−10 °C). Interestingly, the model predicts a Δ*T*_*t*_ with SH3 binding of +7.23 °C, which is also approximately double the DLS experimental measurement of +4 °C. While the predictions for Sjl and Scp(12) are similar, the model predicts essentially no Δ*T*_*t*_ for Prk with SH3 binding, contradicting the observed Δ*T*_*t*_ comparable to those of Scp(12) and Sjl. Given that we used a different ELP backbone, 25 repeats of VPGIG, rather than the VPGXG_90_ (X = V:A:G in a 5:2:3 ratio) backbone used in the development of the Christensen et al. model, and that their model was based on covalent fusions, not equilibrium binding systems, the similarities in our results are encouraging.

**Figure 5:**
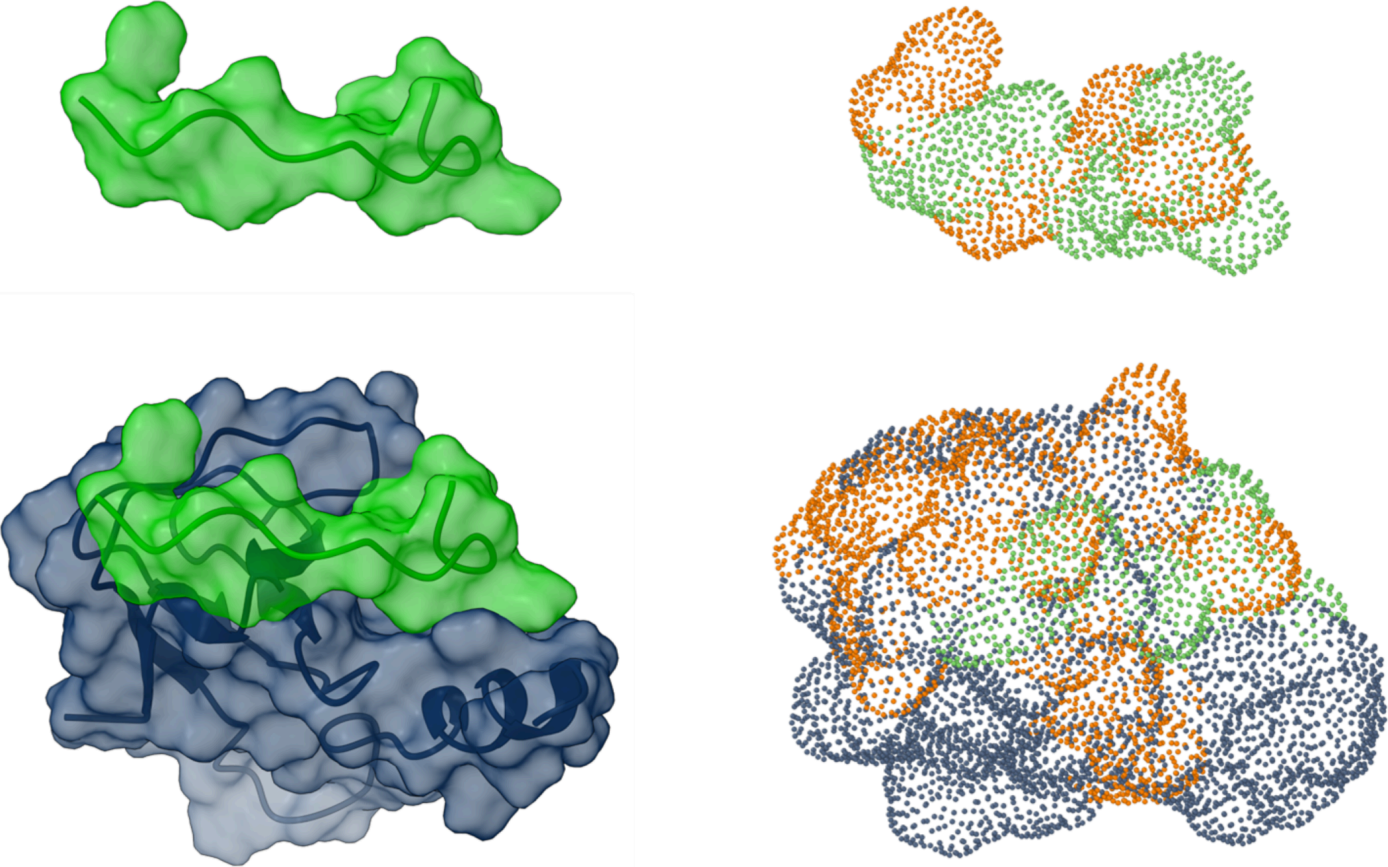
Predicting the complex of Scp(12)-SH3. Predicted structures of Scp(12) (green) alone and in complex with SH3 (blue). Left: space-filling models of the peptide and complex, with semitransparent structures so backbone cartoons are visible. Right: “dots” of the SIC calculations with dots from charged residues in orange.

**Table 2:** Predicted Properties of SH3-Binding ARPs^a^

		Scp(12)	Scp(12)-SH3	Sjl	Sjl-SH3	Prk	Prk-SH3
Asp	Dots	0	578	0	573	0	542
	Dot fraction	0.000	0.094	0.000	0.091	0.000	0.081
	SASA (Å^2^)	0.00	622.39	0.00	607.03	0.00	564.55
	SASA fraction	0.000	0.103	0.000	0.097	0.000	0.087
Glu	Dots	0	423	0	395	209	563
	Dot fraction	0.000	0.069	0.000	0.063	0.081	0.084
	SASA (Å^2^)	0.00	436.92	0.00	425.56	219.94	594.92
	SASA fraction	0.000	0.072	0.000	0.068	0.091	0.091
Lys	Dots	472	712	645	785	406	604
	Dot fraction	0.259	0.116	0.303	0.124	0.157	0.091
	SASA (Å^2^)	423.22	633.70	581.47	710.69	357.56	537.05
	SASA fraction	0.244	0.105	0.292	0.114	0.148	0.082
Arg	Dots	294	318	0	148	592	643
	Dot fraction	0.162	0.052	0.000	0.023	0.228	0.096
	SASA (Å^2^)	284.80	318.02	0.00	146.48	551.59	612.52
	SASA fraction	0.164	0.053	0.000	0.024	0.229	0.094
Total	Dots	1819	6155	2126	6314	2591	6664
	Dot fraction	1.000	1.000	1.000	1.000	1.000	1.000
	SASA (Å^2^)	1737.39	6043.19	1990.84	6230.81	2410.31	6516.14
	SASA fraction	1.000	1.000	1.000	1.000	1.000	1.000
SI_C_		36.68	46.08	31.55	43.40	47.59	47.46
Δ*T*_*t*_ from K-sEL		–19.15	–11.92	–23.10	–13.98	–10.76	–10.86

a Properties of peptides and peptide-SH3 complexes necessary to predict Δ*T*_*t*_ were calculated. The “dots” used in calculating SIC are part of calculating SASA. While the fractions of the dots from each charged residue are similar to the SASA fractions, they are not identical. SIC is calculated using a weighted sum of the dot fractions, and the values were used to predict Δ*T*_*t*_ from K-sEL.

## Conclusions

We have shown that SH3 binding alters the phase transition temperature of SH3-binding peptide-elastin-like polymer fusions, demonstrating that ligand binding can drive distinct structural and phase behaviors. Our findings broadly align with predictions from computational modeling and existing empirical models that relate transition temperature to charged surface area of fusion partners. Predictable ligand-induced shifts in the transition temperature support the feasibility of engineering ARP-based biosensors and adaptive biomaterials. While the characterized system does not itself comprise a biosensor, future studies will explore whether ARPs, like ELPs, can exhibit stimuli-responsive behavior on electrode surfaces detectable by electrochemical impedance for biosensing applications.

## Supplementary Material



## Notes

Supplementary ITC, CD, and DLS data, DNA and protein sequence information, and SDS–PAGE and MS protein characterization (PDF)

## References

[1] Merkx M., Smith B., Jewett M. (2019). Engineering
Sensor Proteins. ACS Sens..

[2] Vallée-Bélisle A., Plaxco K. W. (2010). Structure-switching biosensors: inspired by Nature. Curr. Opin. Struct. Biol..

[3] Lambrianou, A. ; Demin, S. ; Hall, E. A. H. Protein Engineering and Electrochemical Biosensors. In Biosensing for the 21st Century; Renneberg, R. , Lisdat, F. , Eds.; Springer: Berlin, 2008; Vol. 109, pp 65–96. 10.1007/10_2007_080 17960341

[4] Yu E. W., Koshland D. E. (2001). Propagating conformational changes over long (and short)
distances in proteins | PNAS. Proc.Nat.Acad.Sci.98:9517,2001.

[5] Vega S., Abian O., Velazquez-Campoy A. (2016). On the link between conformational
changes, ligand binding and heat capacity. Biochimica
et Biophysica Acta (BBA) - General Subjects.

[6] Grant B. J., Gorfe A. A., McCammon J. A. (2010). Large conformational changes in proteins:
signaling and other functions. Curr. Opin Struct
Biol..

[7] Tsai C., del Sol A., Nussinov R. (2009). Protein allostery,
signal transmission
and dynamics: a classification scheme of allosteric mechanisms. Mol. BioSyst..

[8] Sekhon H., Ha J., Loh S. N. (2023). Enhancing
response of a protein conformational switch
by using two disordered ligand binding domains. Front. Mol. Biosci..

[9] Guo Z., Johnston W. A., Whitfield J., Walden P., Cui Z., Wijker E., Edwardraja S., Retamal Lantadilla I., Ely F., Vickers C., Ungerer J. P. J., Alexandrov K. (2019). Generalizable
Protein Biosensors Based on Synthetic Switch Modules. J. Am. Chem. Soc..

[10] Quijano-Rubio A., Yeh H., Park J., Lee H., Langan R. A., Boyken S. E., Lajoie M. J., Cao L., Chow C. M., Miranda M. C., Wi J., Hong H. J., Stewart L., Oh B., Baker D. (2021). De novo design
of modular and tunable protein biosensors. Nature.

[11] Leonard A. C., Whitehead T. A. (2022). Design and engineering of genetically encoded protein
biosensors for small molecules. Curr. Opin.
Biotechnol..

[12] Broch F., El Hajji L., Pietrancosta N., Gautier A. (2023). Engineering of Tunable
Allosteric-like Fluorogenic Protein Sensors. ACS Sens..

[13] Praetorius F., Leung P. J. Y., Tessmer M. H., Broerman A., Demakis C., Dishman A. F., Pillai A., Idris A., Juergens D., Dauparas J., Li X., Levine P. M., Lamb M., Ballard R. K., Gerben S. R., Nguyen H., Kang A., Sankaran B., Bera A. K., Volkman B. F., Nivala J., Stoll S., Baker D. (2023). Design of stimulus-responsive two-state
hinge proteins. Science.

[14] Meister G. E., Joshi N. S. (2013). An Engineered Calmodulin-Based Allosteric Switch for
Peptide Biosensing. Chembiochem: a European
journal of chemical biology.

[15] Edwardraja S., Guo Z., Whitfield J., Lantadilla I. R., Johnston W. A., Walden P., Vickers C. E., Alexandrov K. (2020). Caged Activators of Artificial Allosteric
Protein Biosensors. ACS Synth. Biol..

[16] Biewenga L., Rosier B. J. H. M., Merkx M. (2020). Engineering with NanoLuc: a playground
for the development of bioluminescent protein switches and sensors. Biochem. Soc. Trans..

[17] Hunt H. K., rmani A. M. (2010). Label-free biological
and chemical sensors. Nanoscale.

[18] Kim B., Chilkoti A. (2008). Allosteric
Actuation of Inverse Phase Transition of
a Stimulus-Responsive Fusion Polypeptide by Ligand Binding. J. Am. Chem. Soc..

[19] Hassouneh W., Nunalee M. L., Shelton M. C., Chilkoti A. (2013). Calcium binding peptide
motifs from calmodulin confer divalent ion selectivity to elastin-like
polypeptides. Biomacromolecules.

[20] Lee J. S., Kang M. J., Lee J. H., Lim D. W. (2022). Injectable Hydrogels
of Stimuli-Responsive Elastin and Calmodulin-Based Triblock Copolypeptides
for Controlled Drug Release. Biomacromolecules.

[21] Trabbic-Carlson K., Meyer D. E., Liu L., Piervincenzi R., Nath N., LaBean T., Chilkoti A. (2004). Effect of protein fusion
on the transition temperature of an environmentally responsive elastin-like
polypeptide: a role for surface hydrophobicity?. Protein Engineering, Design and Selection.

[22] Christensen T., Hassouneh W., Trabbic-Carlson K., Chilkoti A. (2013). Predicting Transition
Temperatures of Elastin-Like Polypeptide Fusion Proteins. Biomacromolecules.

[23] Swartz A. R., Sun Q., Chen W. (2017). Ligand-Induced
Cross-Linking of Z-Elastin-like Polypeptide-Functionalized
E2 Protein Nanoparticles for Enhanced Affinity Precipitation of Antibodies. Biomacromolecules.

[24] Deb B., LaVopa A., McDougal E., Powers J., Denard C., Jang Y. (2025). Recombinant Fusion
Proteins with Embedded Sensing Functions as Versatile
Tools for Protocell Development. Biomacromolecules.

[25] Azagarsamy M. A., Yesilyurt V., Thayumanavan S. (2010). Disassembly of Dendritic Micellar
Containers Due to Protein Binding. J. Am. Chem.
Soc..

[26] Grazon C., Garanger E., Lalanne P., Ibarboure E., Galagan J. E., Grinstaff M. W., Lecommandoux S. (2023). Transcription-Factor-Induced
Aggregation of Biomimetic Oligonucleotide-b-Protein Micelles. Biomacromolecules.

[27] Morales M. A., Paiva W. A., Marvin L., Balog E. R. M., Halpern J. M. (2019). Electrochemical
characterization of the stimuli-response of surface-immobilized elastin-like
polymers. Soft Matter.

[28] Kurochkina N., Guha U. (2013). SH3 domains: modules of protein-protein interactions. Biophys Rev..

[29] Hou T., Xu Z., Zhang W., McLaughlin W. A., Case D. A., Xu Y., Wang W. (2009). Characterization
of domain-peptide interaction interface: a generic
structure-based model to decipher the binding specificity of SH3 domains. Molecular & cellular proteomics.

[30] Saksela K., Permi P. (2012). SH3 domain ligand binding: What’s the consensus and where’s
the specificity?. FEBS Lett..

[31] Kay B. K. (2012). SH3 domains
come of age. FEBS Lett..

[32] Ferreon J. C., Hilser V. J. (2004). Thermodynamics of binding to SH3 domains: the energetic
impact of polyproline II (PII) helix formation. Biochemistry.

[33] Candel A. M., van Nuland N. A. J., Martin-Sierra F. M., Martinez J. C., Conejero-Lara F. (2008). Analysis of
the thermodynamics of binding of an SH3 domain to proline-rich peptides
using a chimeric fusion protein. J. Mol. Biol..

[34] Tian J., Quan J. (2011). Circular polymerase extension cloning for high-throughput cloning
of complex and combinatorial DNA libraries. Nat. Protoc.

[35] Ghosh K., Balog E. R. M., Sista P., Williams D. J., Kelly D., Martinez J. S., Rocha R. C. (2014). Temperature-dependent
morphology
of hybrid nanoflowers from elastin-like polypeptides. APL Mater..

[36] Fazi B., Cope M. J. T. V., Douangamath A., Ferracuti S., Schirwitz K., Zucconi A., Drubin D. G., Wilmanns M., Cesareni G., Castagnoli L. (2002). Unusual binding properties of the
SH3 domain of the yeast actin-binding protein Abp1: structural and
functional analysis. J. Biol. Chem..

[37] Balog E. R. (2023). Periplasmic
Bacterial Expression and Purification of Elastin-like Polymers. protocols.io.

[38] Berman H. M., Westbrook J., Feng Z., Gilliland G., Bhat T. N., Weissig H., Shindyalov I. N., Bourne P. E. (2000). The Protein Data Bank. Nucleic
Acids Res..

[39] Stollar E. J., Garcia B., Chong P. A., Rath A., Lin H., Forman-Kay J. D., Davidson A. R. (2009). Structural, Functional,
and Bioinformatic
Studies Demonstrate the Crucial Role of an Extended Peptide Binding
Site for the SH3 Domain of Yeast Abp1p. J. Biol.
Chem..

[40] Baek M., DiMaio F., Anishchenko I., Dauparas J., Ovchinnikov S., Lee G. R., Wang J., Cong Q., Kinch L. N., Schaeffer R. D., Millán C., Park H., Adams C., Glassman C. R., DeGiovanni A., Pereira J. H., Rodrigues A. V., van Dijk A. A., Ebrecht A. C., Opperman D. J., Sagmeister T., Buhlheller C., Pavkov-Keller T., Rathinaswamy M. K., Dalwadi U., Yip C. K., Burke J. E., Garcia K. C., Grishin N. V., Adams P. D., Read R. J., Baker D. (2021). Accurate prediction
of protein structures and interactions using a three-track neural
network. Science.

[41] Meng E. C., Goddard T. D., Pettersen E. F., Couch G. S., Pearson Z. J., Morris J. H., Ferrin T. E. (2023). UCSF ChimeraX: Tools for structure
building and analysis. Protein Sci..

[42] Huang J., MacKerell A. D. (2013). CHARMM36 all-atom additive protein force field: validation
based on comparison to NMR data. J. Comput.
Chem..

[43] Alford R. F., Leaver-Fay A., Jeliazkov J. R., O’Meara M. J., DiMaio F. P., Park H., Shapovalov M. V., Renfrew P. D., Mulligan V. K., Kappel K., Labonte J. W., Pacella M. S., Bonneau R., Bradley P., Dunbrack R. L. J., Das R., Baker D., Kuhlman B., Kortemme T., Gray J. J. (2017). The Rosetta All-Atom Energy Function for Macromolecular
Modeling and Design. J. Chem. Theory Comput..

[44] Austin K., Torres J. A., Waters J. D. V., Balog E. R. M., Halpern J. M., Pantazes R. J. (2024). An Orthogonal Workflow of Electrochemical, Computational,
and Thermodynamic Methods Reveals Limitations of Using a Literature-Reported
Insulin Binding Peptide in Biosensors. ACS Omega.

[45] Chauhan V. M., Pantazes R. J. (2023). Analysis of conformational stability of interacting
residues in protein binding interfaces. Protein
Eng., Des. Sel..

[46] Islam S., Pantazes R. J. (2023). Developing similarity matrices for antibody-protein
binding interactions. PLoS One.

[47] Richard A. C., Pantazes R. J. (2025). Using Short Molecular Dynamics Simulations to Determine
the Important Features of Interactions in Antibody-Protein Complexes. Proteins.

[48] Lee B., Richards F. M. (1971). The interpretation
of protein structures: Estimation
of static accessibility. J. Mol. Biol..

[49] Shrake A., Rupley J. A. (1973). Environment and
exposure to solvent of protein atoms.
Lysozyme and insulin. J. Mol. Biol..

[50] Urry D. W. (2004). The change
in Gibbs free energy for hydrophobic association: Derivation and evaluation
by means of inverse temperature transitions. Chem. Phys. Lett..

[51] Reguera J., Urry D. W., Parker T. M., McPherson D. T., Rodríguez-Cabello J. C. (2007). Effect of NaCl on the Exothermic
and Endothermic Components of the Inverse Temperature Transition of
a Model Elastin-like Polymer. Biomacromolecules.

[52] Urry D. W. (1988). Entropic
elastic processes in protein mechanisms. II. Simple (passive) and
coupled (active) development of elastic forces. J. Protein Chem..

[53] Reiersen H., Clarke A. R., Rees A. R. (1998). Short elastin-like
peptides exhibit
the same temperature-induced structural transitions as elastin polymers:
implications for protein engineering. J. Mol.
Biol..

