# Supplementary material for: Functional Design and Biophysical Characterization of Analyte-Responsive Polymers
Source: Biomacromolecules. 2025 Jul 25;26(8):4826–37. doi: 10.1021/acs.biomac.5c00066 (PMC12344695; doi:10.1021/acs.biomac.5c00066)

Supporting Information

Title Functional Design and Biophysical Characterization of Analyte-Responsive Polymers

Authors
Carolyn E. Curley,[†] Katarina Jovic Dold,[‡] Jazmine A. Torres,[‖] A. Clay Richard,[‖] Eleenah Sanders,[†] Jeffrey M. Halpern,[‡] Robert J. Pantazes,[‖] Eva Rose M. Balog[†,*]

Affiliations
† School of Molecular and Physical Sciences, University of New England, Biddeford, Maine 04005, United States

‡ Department of Chemical Engineering and Bioengineering, University of New Hampshire, Durham, New Hampshire 03824, United States

‖ Department of Chemical Engineering, Auburn University, Auburn, Alabama 36849, United States

***Corresponding Author:** ebalog@une.edu

Table of Contents for Supporting Information

1. Supplementary Figures and Tables
    - Table S1. Plasmids used in this study.
    - Table S2. Primers used in this study.
    - Table S3. Peptide and protein amino acid sequences.
    - Table S4. SH3-binding peptide sequence alignment.
    - Table S5. DLS-based transition temperatures.
    - Figure S1. Protein purification characterization by SDS-PAGE.
    - Figure S2. Temperature ramp of Scp(12)-sEL prior to SH3 titration.
    - Figure S3: Representative isothermal titration calorimetry data.
    - Figure S4: High-definition mass spectrometry of Scp(12)-sEL protein.
    - Figure S5: Circular dichroism spectra of SH3.
    - Figure S6: Multi-angle dynamic light scattering of individual proteins.

1. Supplementary Figures and Tables

Table S1. Plasmids used in this study. Plasmids are freely available upon request to the corresponding author.

**Plasmid**	**Description**	**Purpose**	**Source or reference**
POE sEL	pelB leader (cleaved upon periplasmic export)<br>C-terminal tags: SV5, 6XHis<br>Subcloning sites: BsshII/NheI<br>Amp resistance	Template for introducing SH3-binding peptide sequences to ELP backbones.	Ghosh et al.[2]
POE K-sEL	pelB leader (cleaved upon periplasmic export)<br>C-terminal tags: SV5, 6XHis<br>Subcloning sites: BsshII/NheI<br>Amp resistance	Control ELP	Balog et al.[17]
POE Scp(12)-sEL	pelB leader (cleaved upon periplasmic export)<br>C-terminal tags: SV5, 6XHis<br>Subcloning sites: BsshII/NheI<br>Amp resistance	Expression of Scp(12)-sEL	This study
POE Sjl-sEL	pelB leader (cleaved upon periplasmic export)<br>C-terminal tags: SV5, 6XHis<br>Subcloning sites: BsshII/NheI<br>Amp resistance	Expression of Sjl-sEL	This study
POE Prk-sEL	pelB leader (cleaved upon periplasmic export)<br>C-terminal tags: SV5, 6XHis<br>Subcloning sites: BsshII/NheI<br>Amp resistance	Expression of Prk-sEL	This study
pET28a SH3	N-terminal tags: 6XHis, thrombin recognition site<br>Subcloning sites: NheI/BamHI<br>Kan resistance	Expression of SH3	This study
pET28a $SH3^{W58A}$	N-terminal tags: 6XHis, thrombin recognition site<br>Subcloning sites: NheI/BamHI<br>Kan resistance	Expression of $SH3^{W58A}$	This study

Table S2. Primers used in this study.

Primers	Purpose	Sequence (5' to 3')
Scp_sEL_F	PCR amplification of double-stranded insert fragment for CPEC	CAAGCGGCGCATGCCGCCGGAcgcccgccggtgaaaagcaaaccgaaacatctgcagggctctggtgtaccaggtatcggtgtccccg
Sjl_sEL_F	PCR amplification of double-stranded insert fragment for CPEC	CAAGCGGCGCATGCCGCCGGAaaaccggaaaaaccgccggtggtgaaaaaaccgcattatctgagcgtggcgggctctggtgtaccaggtatcggtgtccccg
Prk_sEL_F	PCR amplification of double-stranded insert fragment for CPEC	CAAGCGGCGCATGCCGCCGGAaaaagccgcccgccgcgcccgccgccgaaaccgctgcatctgcgcaccgaaggctctggtgtaccaggtatcggtgtccccg
sEL_POE_R	PCR amplification of double-stranded insert fragment for CPEC	GCCCAGCAGTGGGTTTGGGATTGGTTTGCCGCTAGC
POE_inv_F	PCR amplification of double-stranded vector fragment for CPEC	GCTAGCGGCAAACCAATCCCAAACCCACTGCTGGGC (reverse complement of sEL_POE_R to provide overlap)
POE_inv_R	PCR amplification of double-stranded vector fragment for CPEC	CGGCGGCATGCGCGCCGCTTG (reverse complement of capitalized portion of insert F primers to provide overlap)

Table S3. Peptide and protein amino acid sequences.

Peptides	Purpose	Sequence	Molecular Weight (kDa)
Scp(12)	ITC experiments	RPPVKSKPKHLQ	1414.72
Prk	ITC experiments	KSRPPRPPPKPLHLRTE	2006.38
**Proteins**	**Sequence**		
Scp(12)-sEL	AG**RPPVKSKPKHLQ**GSG[VPGIG]$_{25}$VPASGKPIPNPLLGLDSTHHHHHH		14912.74
K-sEL	AG**K**GSG[VPGIG]$_{25}$VPASGKPIPNPLLGLDSTHHHHHH		13644.21
Sjl-sEL	AG**KPEKPPVVKKPHYLSVA**GSG[VPGIG]$_{25}$VPASGKPIPNPLLGLDSTHHHHHH		15415.34
Prk-sEL	AG**KSRPPRPPPKPLHLRTE**GSG[VPGIG]$_{25}$VPASGKPIPNPLLGLDSTHHHHHH		15504.40
SH3	MGSSHHHHHHSSGLVPRGSHMASPWATAEYDYDAAEDNELTFVENDKIINIEFVDDDWWLGELEKDGSKGLFPSNYVSLGN		9112.82
SH3^{W58A}	MGSSHHHHHHSSGLVPRGSHMASPWATAEYDYDAAEDNELTFVENDKIINIEFVDDD**A**WLGELEKDGSKGLFPSNYVSLGN		8997.68

Table S4. SH3-binding peptide sequence alignment

Peptide	Sequence
Ark1p	AKKTKPTPPPKPSHLKPK
Scp(12)	----R**P**PVKS**KP**K**HL**Q--
Sjl	----**KP**PVVK**KP**HY**L**SVA
Prk	-**K**SRP**P**R**PPPKP**L**HL**RTE

Table S5. DLS-based transition temperatures. Temperatures at which particle size exceeds 200 nm for each condition.

**Condition**	**T_t (°C) Rep 1**	**T_t (°C) Rep 2**	**Mean**	**SD**
Scp(12)-sEL	18	18	18	-
Scp(12)-sEL + SH3	22	22	22	-
K-sEL	28	26	27	±1.4
K-sEL + SH3	28	28	28	-
Scp(12)-sEL + $SH3^{W58A}$	16	16	16	-
Scp(12)-sEL, 200 mM NaCl	16	18	17	±1.4
Scp(12)-sEL + SH3, 200 mM NaCl	20	20	20	-
K-sEL, 200 mM NaCl	18	18	18	-
K-sEL + SH3, 200 mM NaCl	18	18	18	-
Sjl-sEL*	8	14	12	±4.2
Sjl-sEL + SH3*	12	14	13	±1.4
Prk-sEL	14	18	16	±2.8
Prk-sEL + SH3	18	20	19	±1.4
Scp(12)-sEL in MEMα*†	34	-	34	-
Scp(12)-sEL + SH3 in MEMα*†	50	-	50	-
K-sEL in MEMα*	26	-	26	
K-sEL + SH3 in MEMα*	24	-	24	

*Lower threshold (100 nm) applied as assemblies in one of the compared conditions (±SH3) remained below 600 nm.

†Measured once to confirm UV-Vis results

Figure S1. SDS-PAGE analyses of recombinant protein purification. (A) *E. coli* expressing His-tagged SH3 were subjected to lysis and clarification by centrifugation. Supernatant was applied to a Ni-NTA column. Flow through, wash (8 column volumes total), and elution (5 mL x 8) fractions were analyzed. 6xHis-SH3 = 9.1 kDa and routinely runs slightly higher. (B), (C), (E), and (F) Hypertonic and hypotonic fractions of the periplasmic extraction procedure, supernatant after precipitation with 3 M NaCl, and the cold supernatant after each round of inverse temperature cycling were analyzed. (D) *E. coli* expressing His-tagged SH3^{W58A} were subjected to lysis and clarification by centrifugation. Supernatant was applied to a Ni-NTA column. Supernatant and pellet fractions following clarification, flow through, wash, and 6 elution fractions were analyzed. Distorted appearance in several elution fraction samples is due to overloading of the gel—the final elution lane appears normal. The whole unaltered gel image is shown for transparency; a different purified protein is shown in the unlabeled lane adjacent to the ladder. 6XHis-SH3^{W58A} = 9.0 kDa and routinely runs slightly higher.

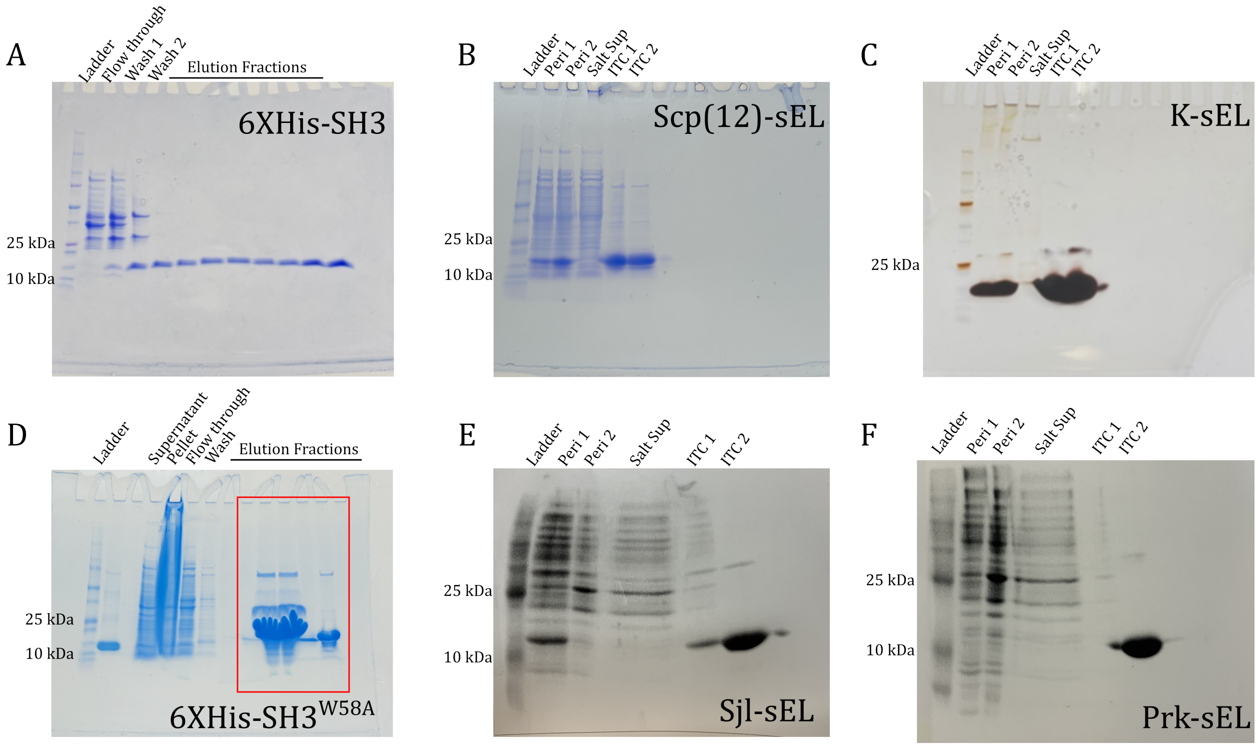

Figure S2. Temperature ramp of Scp(12)-sEL prior to SH3 titration.

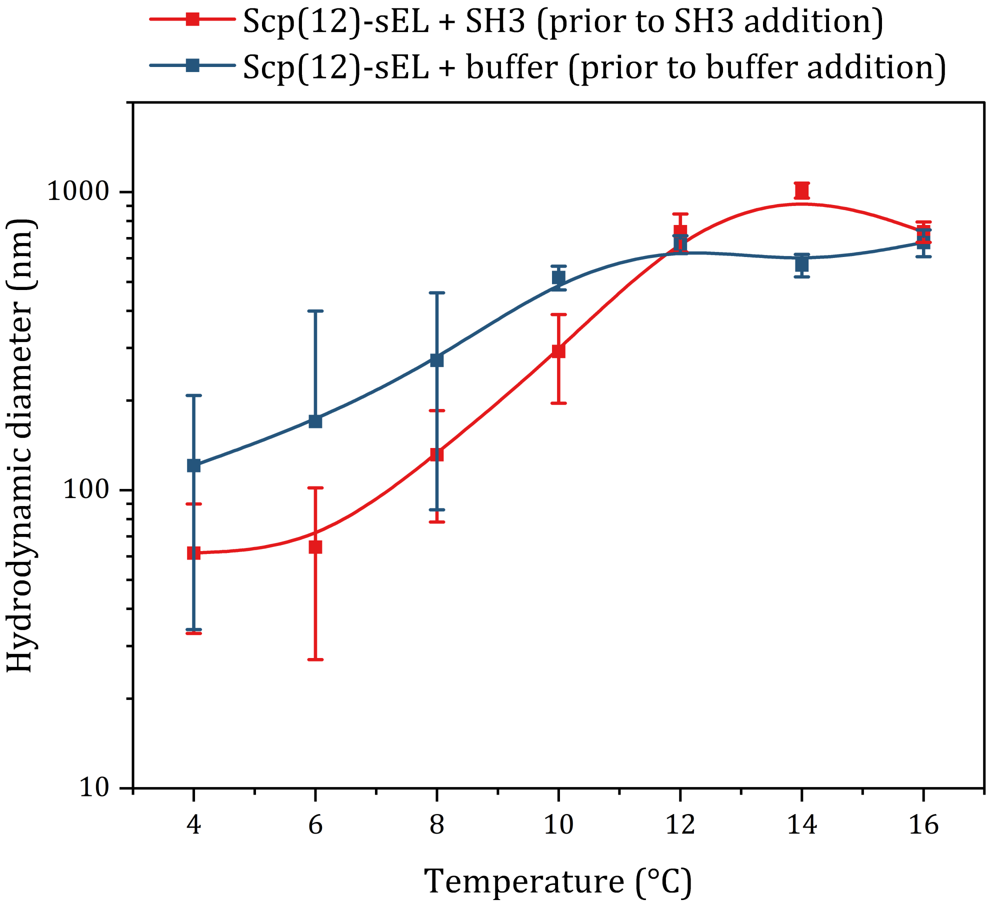

Figure S3. Representative isothermal titration calorimetry data.

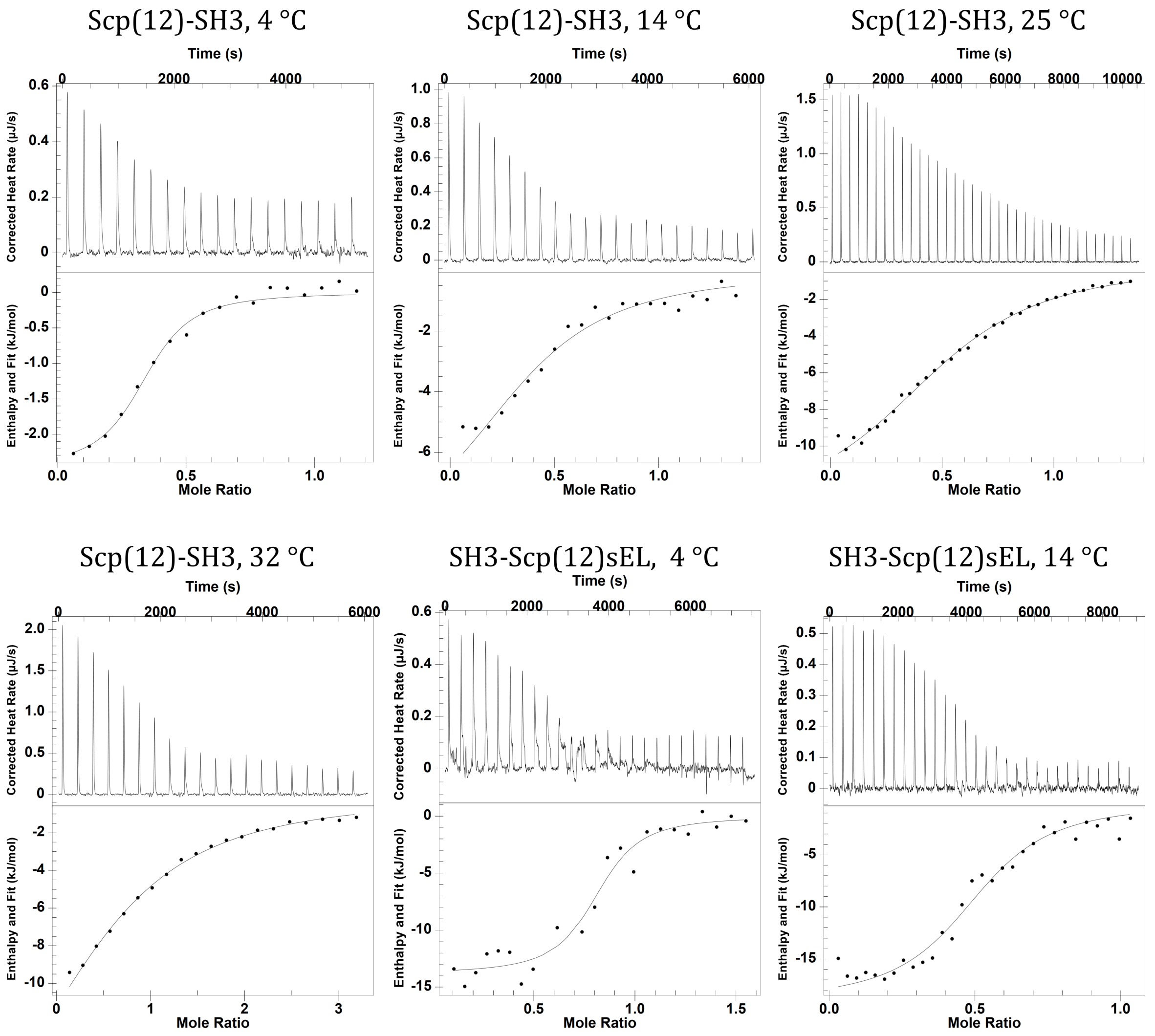

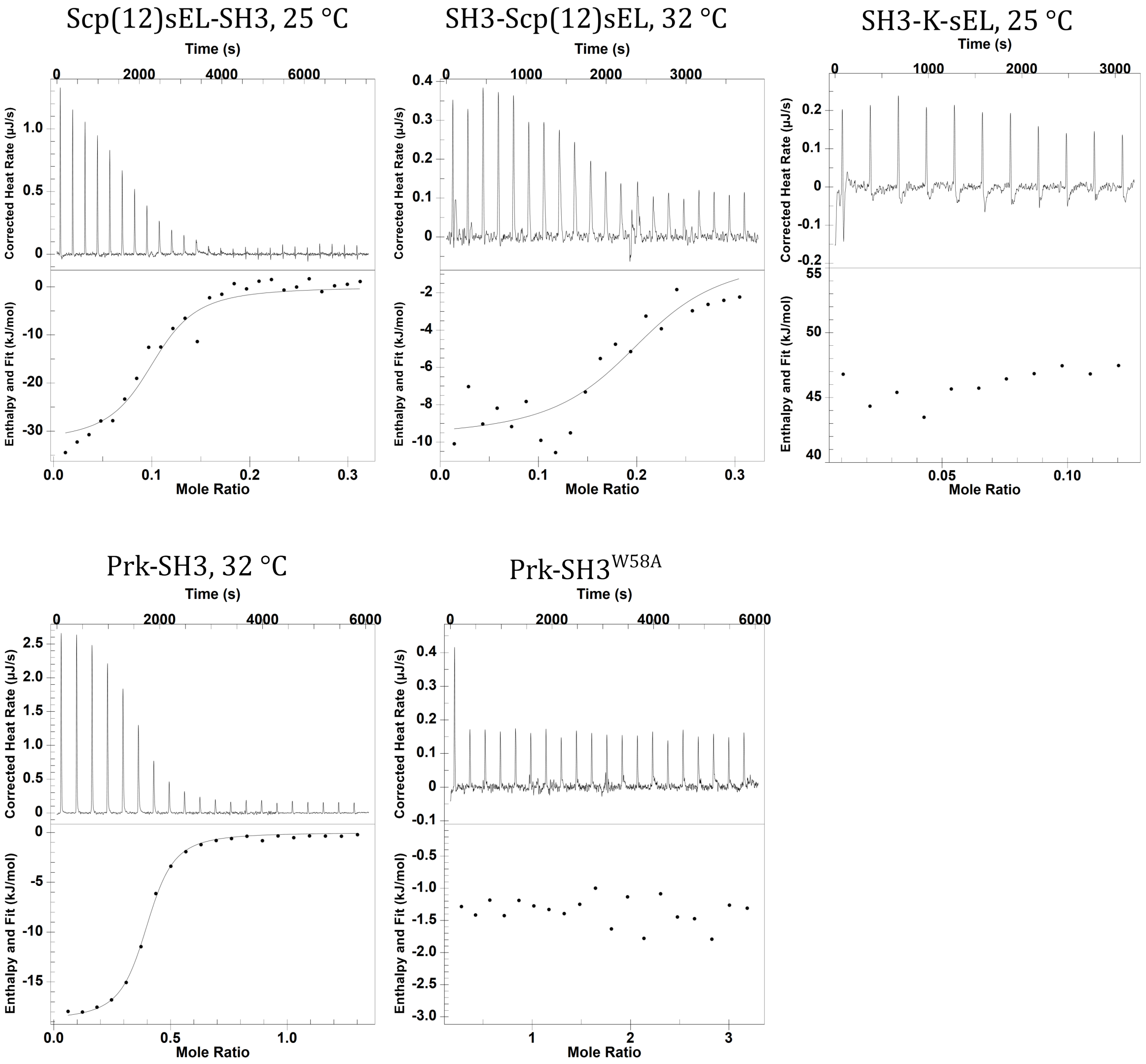
Scp(12)sEL-SH3, 25 °C
SH3-Scp(12)sEL, 32 °C
SH3-K-sEL, 25 °C
Prk-SH3, 32 °C
Prk-SH3^W58A
Time (s)
Corrected Heat Rate (µJ/s)
Enthalpy and Fit (kJ/mol)
Mole Ratio

Figure S4. Mass spectrometry confirms the expected molecular weight of Scp(12)-sEL. The calculated molecular weight of Scp(12)-sEL based on its amino acid sequence is 14912.74.

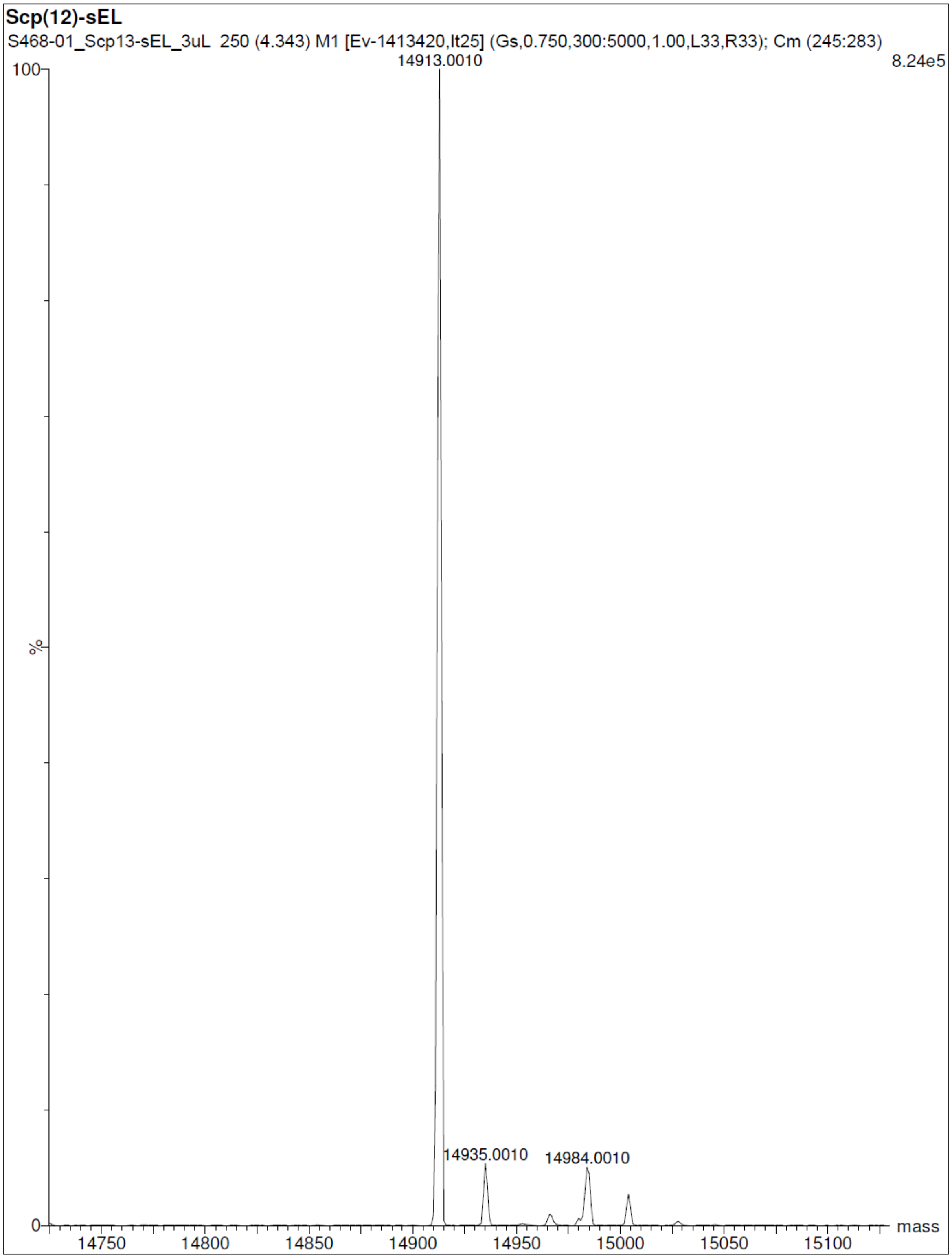

Figure S5. Circular dichroism spectra of SH3.

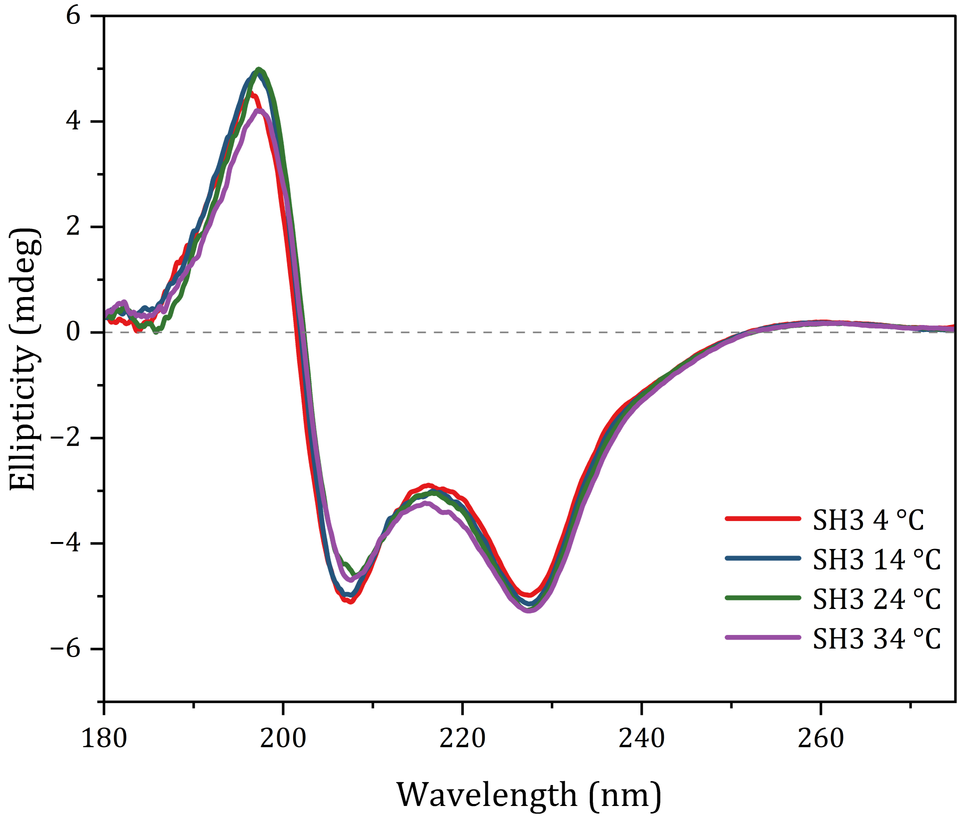

Figure S6. Multi-angle dynamic light scattering (MADLS). MADLS provides accurate particle size distributions for SH3 (top), Scp(12)-sEL (middle), and K-sEL (bottom) at three different temperatures.

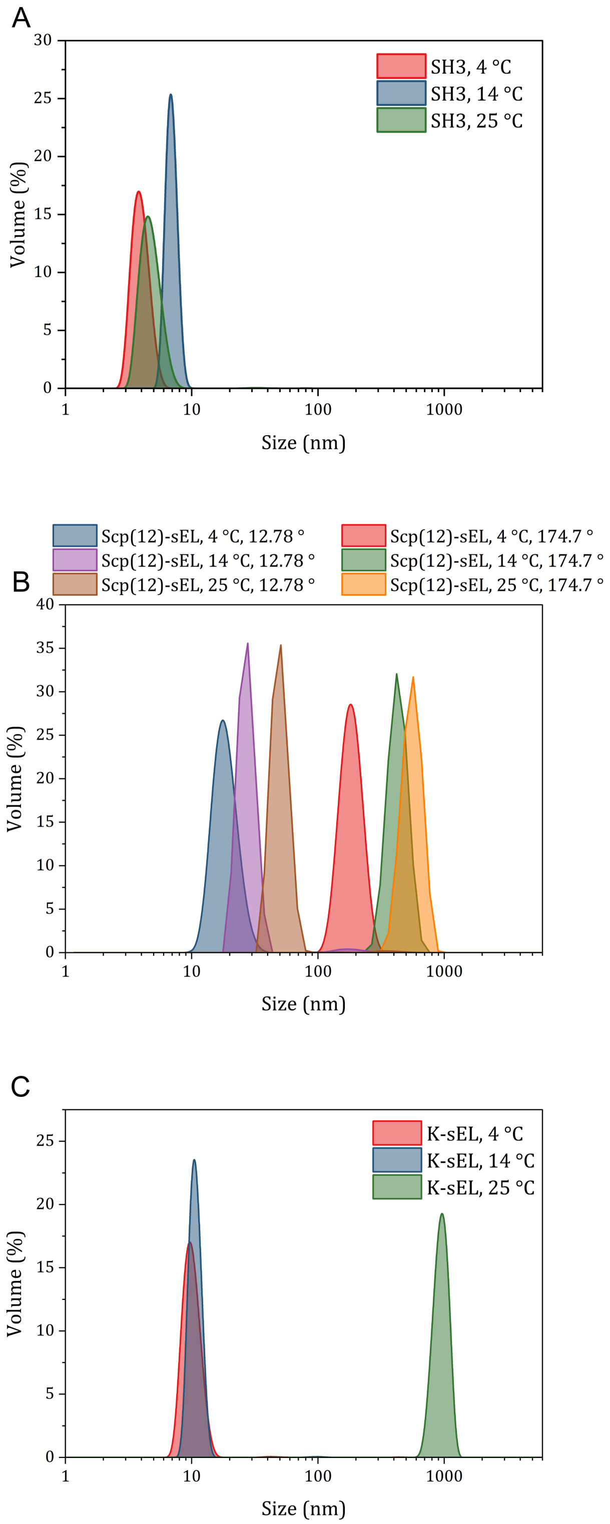

Supplement: Supplementary file 1 [file bm5c00066_si_001.pdf]
